# A comparison of the performance of seven key bibliographic databases in identifying all relevant systematic reviews of interventions for hypertension

**DOI:** 10.1186/s13643-016-0197-5

**Published:** 2016-02-09

**Authors:** John Rathbone, Matt Carter, Tammy Hoffmann, Paul Glasziou

**Affiliations:** Centre for Research in Evidence Based Practice, Bond University, Gold Coast, Australia

**Keywords:** EMBASE, MEDLINE, PubMed Health, TRIP, DARE, Epistemonikos, The Cochrane library, Systematic review, Evaluation

## Abstract

**Background:**

Bibliographic databases are the primary resource for identifying systematic reviews of health care interventions. Reliable retrieval of systematic reviews depends on the scope of indexing used by database providers. Therefore, searching one database may be insufficient, but it is unclear how many need to be searched. We sought to evaluate the performance of seven major bibliographic databases for the identification of systematic reviews for hypertension.

**Methods:**

We searched seven databases (Cochrane library, Database of Abstracts of Reviews of Effects (DARE), Excerpta Medica Database (EMBASE), Epistemonikos, Medical Literature Analysis and Retrieval System Online (MEDLINE), PubMed Health and Turning Research Into Practice (TRIP)) from 2003 to 2015 for systematic reviews of any intervention for hypertension. Citations retrieved were screened for relevance, coded and checked for screening consistency using a fuzzy text matching query. The performance of each database was assessed by calculating its sensitivity, precision, the number of missed reviews and the number of unique records retrieved.

**Results:**

Four hundred systematic reviews were identified for inclusion from 11,381 citations retrieved from seven databases. No single database identified all the retrieved systematic reviews for hypertension. EMBASE identified the most reviews (sensitivity 69 %) but also retrieved the most irrelevant citations with 7.2 % precision (Pr). The sensitivity of the Cochrane library was 60 %, DARE 57 %, MEDLINE 57 %, PubMed Health 53 %, Epistemonikos 49 % and TRIP 33 %. EMBASE contained the highest number of unique records (*n* = 43). The Cochrane library identified seven unique records and had the highest precision (Pr = 30 %), followed by Epistemonikos (*n* = 2, Pr = 19 %). No unique records were found in PubMed Health (Pr = 24 %) DARE (Pr = 21 %), TRIP (Pr = 10 %) or MEDLINE (Pr = 10 %). Searching EMBASE and the Cochrane library identified 88 % of all systematic reviews in the reference set, and searching the freely available databases (Cochrane, Epistemonikos, MEDLINE) identified 83 % of all the reviews.

The databases were re-analysed after systematic reviews of non-conventional interventions (e.g. yoga, acupuncture) were removed. Similarly, no database identified all the retrieved systematic reviews. EMBASE identified the most relevant systematic reviews (sensitivity 73 %) but also retrieved the most irrelevant citations with Pr = 5 %. The sensitivity of the Cochrane database was 62 %, followed by MEDLINE (60 %), DARE (55 %), PubMed Health (54 %), Epistemonikos (50 %) and TRIP (31 %). The precision of the Cochrane library was the highest (20 %), followed by PubMed Health (Pr = 16 %), DARE (Pr = 13 %), Epistemonikos (Pr = 12 %), MEDLINE (Pr = 6 %), TRIP (Pr = 6 %) and EMBASE (Pr = 5 %). EMBASE contained the most unique records (*n* = 34). The Cochrane library identified seven unique records. The other databases held no unique records.

**Conclusions:**

The coverage of bibliographic databases varies considerably due to differences in their scope and content. Researchers wishing to identify systematic reviews should not rely on one database but search multiple databases.

## Background

Systematic reviews provide the best evidence of the effects of health care interventions [1]. However, identifying systematic reviews can be time-consuming and haphazard because no database covers all health topics [2]. Therefore, searching several databases is a necessity when seeking health research, including systematic reviews. With the growth [3] and scatter of research [4], finding relevant and up-to-date information is becoming increasingly difficult. Moreover, clinicians who perform quick clinical queries with one database often lack the training and skills to run efficient searches and subsequently produce imprecise results [5]. Understandably, there is currently no specific guidance on which databases should be searched to find systematic reviews, only general advice to search widely. For example, researchers planning a systematic review are recommended to first search for existing reviews which answer the research question to avoid duplicating research [6], but it is unclear which is the best database to search or how many should be searched.

The aim of this study was to evaluate seven databases—the Cochrane library, the Database of Abstracts of Reviews of Effects (DARE), Excerpta Medica Database (EMBASE), Epistemonikos, Medical Literature Analysis and Retrieval System Online (MEDLINE), PubMed Health and Turning Research Into Practice (TRIP)—to determine their coverage of systematic reviews assessing effectiveness of interventions of a typical high-prevalence condition, hypertension, and to determine how many databases require searching to identify all relevant systematic reviews.

## Methods

**Fig. 1:**
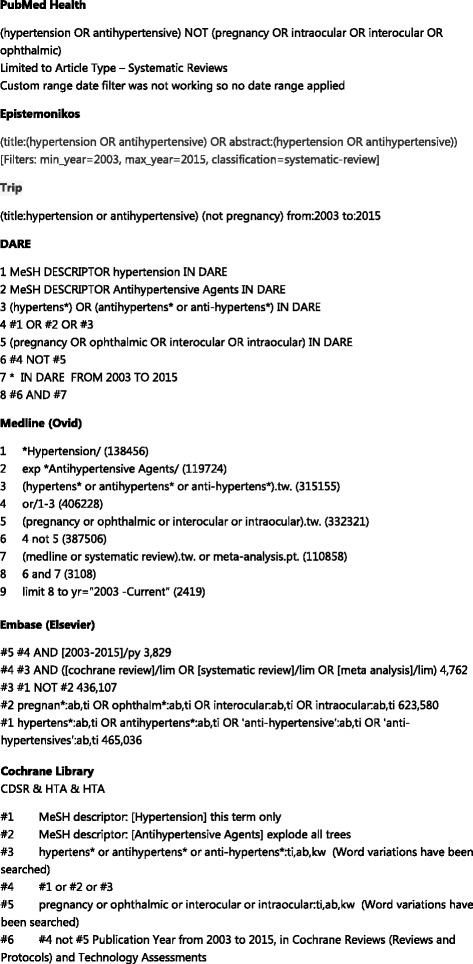
Search strategies

We searched seven databases (EMBASE, MEDLINE, the Cochrane library (inc. CDSR, DARE and HTA), Epistemonikos, PubMed Health, DARE and TRIP) for systematic reviews of any treatment interventions for hypertension from 2003 to Jan 2015 (see Fig. 1). We used an open definition of systematic review which included reviews stated or described as being a systematic review or meta-analysis. Reports and summaries of evidence were excluded. PICO criteria were defined as follows: participants, i.e. people with hypertension by any definition; interventions, any; comparator, any; and outcomes, change in blood pressure. Systematic review filterers incorporated into the databases were selected to increase search sensitivity. For MEDLINE, we used the Montori filter [7]. Citations retrieved were imported into separate EndNote X7 libraries, and then titles and abstracts were screened for relevance by one reviewer. Reviews of pre-hypertension, ophthalmic, pulmonary, pregnancy-related hypertension or hepatic hypertension were excluded.

Citations were coded in EndNote X7 as either a systematic review or not. Screening decisions in one database were cross-checked against the other six databases to ensure consistency using a title-matching database query. The query incorporated a fuzzy text matching algorithm [8, 9] to account for differences with punctuation or syntax errors. Where screening decisions were found to be inconsistent, these were re-examined and standardised across the databases. Where databases (e.g. PubMed Health) used the Cochrane plain language title rather than the original full title, these were changed to the full title for consistency with other databases.

### Data analysis

The performance of each database was assessed by calculating the sensitivity (number of relevant studies/reference set × 100); the precision (number of relevant studies/number of studies retrieved × 100); the number missed (reference set − number of relevant studies); and the number of unique records, i.e. records only found in one database. The reference set is the total of unique systematic reviews identified across all the databases. Records identified as being unique were double-checked for accuracy using a title search within the (online) comparator bibliographic databases without the systematic review search filters applied. A secondary analysis was performed by removing all non-conventional treatments, i.e. systematic reviews that are not prescribed drugs, e.g. yoga, acupuncture, herbal medicine, and exercise programmes, from the databases and re-calculated to provide results reflecting the type of quick clinical queries clinicians would run.

## Results

**Fig. 2:**
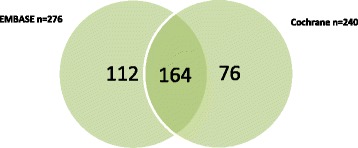
Proportion of reference set (*n* = 400) retrieved by searching *EMBASE* and the *Cochrane* library, resulting in the identification of 88 % (*n* = 352) of total reviews

**Fig. 3:**
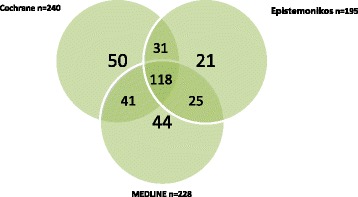
Proportion of reference set (*n* = 400) retrieved by searching *Cochrane*, *Epistemonikos* and *MEDLINE*, resulting in the identification of 83 % (*n* = 330) of total reviews

There were 400 systematic reviews (the reference set) identified for inclusion from a total of 11,381 citations retrieved from seven databases. No database identified all 400 included systematic reviews of interventions for hypertension (Table 1). EMBASE retrieved the highest number of relevant reviews (*n* = 276) with a sensitivity (*s*) of 69.0 %, followed by Cochrane (*n* = 240, *s* = 60.0 %), DARE (*n* = 228, *s* = 57.0 %), MEDLINE (*n* = 228, *s* = 57.0 %), PubMed Health (*n* = 212, *s* = 53.0 %), Epistemonikos (*n* = 195, *s* = 48.8 %) and TRIP (*n* = 131, *s* = 32.8 %). EMBASE contained the largest number of unique records (*n* = 43) but had the lowest precision (Pr, 7.2 %). Cochrane contained seven unique records and had the highest precision (29.9 %), followed by Epistemonikos (*n* = 2, Pr = 19.2 %). No unique records were found in PubMed Health (Pr = 23.6 %), DARE (Pr = 20.8 %), TRIP (Pr = 9.7 %) or MEDLINE (Pr = 9.6 %). Searching the two databases with the highest sensitivity and unique records (EMBASE and the Cochrane library) identified 88 % of the reference set (Fig. 2). Searching the Cochrane library, MEDLINE and Epistemonikos identified 83 % of the reference set (Fig. 3).

**Table 1:** Performance of bibliographic databases identifying relevant systematic reviews of interventions for treating hypertension

Database	Reviews relevant	Reviews missed	Total citations	Sensitivity (*s*) %	Precision (Pr) %	Unique records
EMBASE	276	124	3836	69.0	7.2	43
Cochrane	240	160	802	60.0	29.9	7
DARE	228	172	1098	57.0	20.8	0
MEDLINE	228	172	2374	57.0	9.6	0
PubMed Health	212	188	899	53.0	23.6	0
Epistemonikos	195	205	1017	48.8	19.2	2
TRIP	131	269	1355	32.8	9.7	0

Reference set of included systematic review (*n* = 400)

After removing 168 non-conventional medical interventions for hypertension, e.g. yoga, acupuncture, herbal medicine, and exercise programmes, there were 232 systematic reviews remaining in the reference set. Again, no database identified all included systematic reviews of conventional interventions for hypertension (Table 2). EMBASE retrieved the highest number of relevant records (*n* = 169) with a sensitivity of 72.8 %, followed by the Cochrane library (*n* = 143, *s* = 61.6 %), MEDLINE (*n* = 138, *s* = 59.5 %), DARE (*n* = 127, *s* = 54.7 %), PubMed Health (*n* = 126, *s* = 54.3 %), Epistemonikos (*n* = 116, *s* = 50.0 %) and TRIP (*n* = 72, *s* = 31.0 %). EMBASE contained the largest number of unique records (*n* = 34) but had the lowest precision (Pr = 4.5 %). Cochrane contained seven unique records and had the highest precision (Pr = 20.3 %). No unique records were found in PubMed Health (Pr = 15.5 %), DARE (Pr = 12.7 %), Epistemonikos (Pr = 12.4 %), MEDLINE (Pr = 6.0 %) or TRIP (Pr = 5.5 %).

**Table 2:** Performance of bibliographic databases identifying relevant systematic reviews of interventions for treating hypertension (excluding non-conventional treatments)

Database	Reviews relevant	Reviews missed	Total citations	Sensitivity (*s*) %	Precision (Pr) %	Unique records
EMBASE	169	63	3722	72.8	4.5	34
Cochrane	143	89	704	61.6	20.3	7
MEDLINE	138	94	2282	59.5	6.0	0
DARE	127	105	998	54.7	12.7	0
PubMed Health	126	106	812	54.3	15.5	0
Epistemonikos	116	116	938	50.0	12.4	0
TRIP	72	160	1320	31.0	5.5	0

Reference set of included systematic review (*n* = 232)

## Discussion

Seven databases were searched—the Cochrane library, DARE, EMBASE, Epistemonikos, MEDLINE, PubMed Health and TRIP—to determine their coverage of systematic reviews of interventions for hypertension. No single database retrieved the entire reference set of 400 reviews; EMBASE had the highest sensitivity of 69 % but would still miss 124 reviews. Searching both the Cochrane library and EMBASE identified 88 % of the reference set. EMBASE, however, is a subscription service and many institutions do not subscribe to EMBASE, which may limit some clinicians from performing clinical queries. Nevertheless, in the example used in this study, searching the Cochrane library, MEDLINE and Epistemonikos retrieves 83 % of the reference set.

Our findings have illustrated that despite the broad scope of many bibliographic databases, relying on one or two to identify a systematic review is not always possible, and wider search should be considered to ensure systematic reviews are not missed.

### Strengths and limitations

We used systematic review filters to increase precision during the search for hypertension reviews, which can reduce the sensitivity. Therefore, records classed as unique were cross-checked with the comparator databases by searching in title fields without applying the filter to ensure the record was genuinely unique rather than missed due to filtering. However, this procedure was not performed where systematic reviews were found in two or more databases, and therefore, some reviews may have been missed due to use of filters or the reviews being inadequately coded in the databases. Screening was performed by one reviewer with the potential for screening errors between databases; therefore, to ensure screening decisions were consistent, a fuzzy text matching query [10] was used. Our case study did not include every bibliographic database available, but we included seven major databases, including the two largest (EMBASE and MEDLINE); however, the results may not be applicable to specialist databases if they are not indexed in MEDLINE, EMBASE or the Cochrane library. Our focus was limited to one clinical condition (hypertension), but other clinical topics are also likely to be dispersed throughout these databases without a single database containing all records. Other study designs such as prognostic and diagnostic studies were not evaluated, and database searches for this type of study design may perform differently. The DARE database provided a search platform with good overall sensitivity and precision, but funding for DARE ceased at the end of March 2015 ([11]), and as it is no longer being updated, this database will increasingly become less sensitive for identifying systematic reviews.

## Conclusions

This case study demonstrated that relying on a single database is insufficient to identify all relevant systematic reviews. Depending on the database used, the chances of finding a systematic review topic range from 33 to 69 %, and therefore, searching should not be restricted to two major databases; instead, a search of all databases should be performed to determine if a review title exists. Further research is warranted to assess how these findings might extend to other topic areas and study designs.

## Abbreviations

CDSR: Cochrane database of systematic reviews
DARE: Database of abstracts of reviews of effects
EMBASE: Excerpta medica database
HTA: Health technology appraisal
MEDLINE: Medical literature analysis and retrieval system online
PICO: Participants, interventions, comparators, outcomes
TRIP: Turning research into practice
